# Postbiotic Nagqu4580 Attenuates Ulcerative Colitis and Suppresses Ferroptosis in Association with the Microbiota-Tryptophan-AhR/Nrf2 Axis

**DOI:** 10.3390/nu18132150

**Published:** 2026-07-02

**Authors:** Xiangjun Chen, Zhengyang Hao, Ruipeng Wu, Huan Zhang, Siying Tu, Shaokang Wang, Guiju Sun

**Affiliations:** 1 Key Laboratory of Environmental Medicine and Engineering of Ministry of Education, Department of Nutrition and Food Hygiene, School of Public Health, Southeast University, Nanjing 210009, China; xjchen@xmzu.edu.cn (X.C.); wurp@xzmu.edu.cn (R.W.); huanzhang@163.com (H.Z.); 230248566@seu.edu.cn (S.T.); gjsun@seu.edu.cn (G.S.); 2 Clinical Medical Research Center for Plateau Gastroenterological Disease of Xizang Autonomous Region, School of Medicine, Xizang Minzu University, Xianyang 712082, China; haozy6@163.com

**Keywords:** ferroptosis, ulcerative colitis, postbiotic Nagqu4580, tryptophan metabolism, AhR/Nrf2

## Abstract

**Background/Objectives**: Ferroptosis, an iron-dependent cell death driven by lipid peroxidation, is implicated in the pathogenesis of ulcerative colitis (UC). Tryptophan metabolism and its interaction with the aryl hydrocarbon receptor (AhR) and nuclear factor erythroid 2–related factor 2 (Nrf2) axis represent a crucial regulatory network in intestinal homeostasis. This study aimed to investigate whether the probiotic fermentation product postbiotic Nagqu4580 alleviates UC by modulating this network to inhibit intestinal epithelial ferroptosis. **Methods**: An acute UC model was induced in mice using 4% dextran sodium sulfate (DSS). The therapeutic effects of postbiotic Nagqu4580 were evaluated through disease activity index (DAI), colon length, histopathology, inflammatory cytokines, and intestinal barrier function. Ferroptosis was assessed by measuring lipid peroxidation (MDA, 4-HNE), antioxidant capacity (GSH/GSSG), and expression levels of GPX4 and ACSL4. Serum tryptophan metabolites were profiled using targeted metabolomics, the activation of the AhR/Nrf2 pathway was examined by Western blot, immunofluorescence, and qPCR, and gut microbiota composition was analyzed by 16S rRNA sequencing. **Results**: Postbiotic Nagqu4580 dose-dependently ameliorated DSS-induced UC in mice, as evidenced by reduced DAI scores, mitigated colon shortening and histological damage, decreased inflammatory cytokines (TNF-α, IL-1β, IL-6), and restored intestinal barrier function by upregulating tight junction proteins (Claudin-1, ZO-1, Occludin). Mechanistically, postbiotic Nagqu4580 inhibited intestinal epithelial ferroptosis by reducing MDA and 4-HNE levels, restoring the GSH/GSSG balance, downregulating ACSL4, and upregulating GPX4. Serum metabolomics revealed that postbiotic Nagqu4580 reshaped tryptophan metabolism, increasing beneficial metabolites such as 5-hydroxyindoleacetic acid (5-HIAA) and decreasing potentially harmful metabolites such as 3-indoxyl sulfate (3-IS). 16S rRNA sequencing further revealed that the postbiotic Nagqu4580 partially reversed DSS-induced gut microbiota dysbiosis, with a slight increase in the abundance of beneficial genera and a significant reduction in the abundance of pro-inflammatory genera. Furthermore, postbiotic Nagqu4580 significantly activated the AhR/Nrf2 signaling pathway, enhancing the expression of AhR, Nrf2, and their downstream antioxidant genes HO-1 and GPX4. **Conclusions**: Postbiotic Nagqu4580 alleviates UC by inhibiting intestinal epithelial ferroptosis. Our data suggest that this protective effect is associated with the remodeling of gut microbiota-related tryptophan metabolism and subsequent activation of the AhR/Nrf2 antioxidant axis. Our findings highlight the therapeutic potential of postbiotic Nagqu4580 as a postbiotic agent for UC.

## 1. Introduction

Ferroptosis is an iron-dependent form of regulated cell death, characterized by dysregulated iron metabolism, lipid peroxidation, and glutathione (GSH) depletion [1]. In recent years, substantial evidence has established that ferroptosis plays a significant role in the pathogenesis of ulcerative colitis (UC), particularly in relation to intestinal inflammatory responses and intestinal epithelial cell (IEC) damage. Ferroptosis inhibitors (such as Ferrostatin-1, Fer-1) have been shown to significantly alleviate inflammatory responses and intestinal epithelial injury in animal and cellular models [2,3,4]. Furthermore, regulating iron metabolism (e.g., reducing iron load), decreasing lipid peroxidation, and maintenance GSH levels are important therapeutic strategies for mitigating UC symptoms [5,6].

Tryptophan, an essential amino acid, has garnered increasing attention for its role in intestinal inflammation, especially in the pathogenesis of inflammatory bowel disease (IBD), including Crohn’s disease and ulcerative colitis. Gut microbiota in patients with IBD is characterized by a reduction in beneficial bacteria (such as Faecalibacterium and Ruminococcus) and an increase in harmful bacteria. This dysbiosis alters the production of tryptophan metabolites, including a decrease in indole analogs, thereby compromising immune regulatory functions mediated through the aryl hydrocarbon receptor (AhR) pathway [7,8,9]. Activation of AhR induces the production of anti-inflammatory factors like interleukin-22 (IL-22), thus helping to maintain intestinal barrier integrity. A reduction in tryptophan metabolites, such as 3-IAld, may exacerbate the inflammatory state in IBD [10,11,12]. Experimental evidence has shown that modulating tryptophan metabolism, for instance, via Ganoderic acid A, can improve intestinal barrier function and alleviate colonic inflammation, which further supports the protective role of tryptophan metabolites in IBD [13,14].

Tryptophan metabolism is critically involved in regulating ferroptosis. Its metabolites can either inhibit or promote ferroptotic processes through multiple mechanisms. Research indicates that tryptophan metabolites like serotonin (5-HT) and 3-hydroxyanthranilic acid (3-HA) can act as radical-trapping antioxidants (RTAs), directly scavenge lipid peroxidation products and thereby inhibit ferroptosis [15]. Similarly, the tryptophan metabolism enzyme IL4I1 reduces reactive oxygen species (ROS) accumulation by producing indole-3-pyruvate (I3P) and inhibits the ubiquitination and degradation of Nrf2 through binding, which upregulates antioxidant gene expression and subsequently suppresses ferroptosis [16,17,18,19]. Furthermore, 3-HA can directly bind to ferritin heavy chain (FTH1) and interfere with its interaction with nuclear receptor coactivator 4 (NCOA4), thereby inhibiting ferritinophagy, reducing the release of free iron, and ultimately alleviating ferroptosis [20]. Conversely, the regulation of tryptophan metabolism can also affect sensitivity to ferroptosis. For instance, tryptophan itself can inhibit ferroptosis by suppressing NCOA4-mediated FTH1 autophagy and stabilizing ferritin levels [21].

Additionally, the activation of AhR signaling, associated with tryptophan and its metabolites, plays an essential role in inhibiting ferroptosis. This process typically involves the activation of the Nrf2 (nuclear factor erythroid 2-related factor 2) antioxidant pathway. In sepsis-induced acute kidney injury, the AhR agonist FICZ promotes the nuclear translocation of AhR and Nrf2, upregulates the expression of GPX4 and SLC7A11, reduces lipid peroxide accumulation, and thereby inhibits ferroptosis [22]. In models of doxorubicin-induced cardiomyopathy and ischemic stroke, the microbial metabolite indole-3-lactic acid (ILA), acting as an AhR ligand, inhibits ferroptosis in cardiomyocytes and neurons by activating the AhR/Nrf2 signaling axis and upregulating SLC7A11 and GPX4 [19,23]. In lung cancer cells, IDO1 activates the AhR/Nrf2 axis, upregulates SLC7A11, enhances the pentose phosphate pathway (PPP) activity, promotes the generation of NADPH and GSH, and thus confers resistance to Erastin-induced ferroptosis [24]. Moreover, pharmacological inhibition or genetic ablation of AhR has been shown to enhance Erastin-induced ferroptosis. This mechanism is associated with AhR-mediated transcriptional regulation of solute carrier family genes like SLC7A11, while the endogenous ligand indole-3-pyruvate (I3P) protects cells from ferroptosis in an AhR-dependent manner [25].

In recent years, microecological preparations, including probiotics, prebiotics, and postbiotics, have attracted increasing attention for their beneficial effects on gastrointestinal inflammation, particularly in the context of IBD [26]. Probiotics are defined as live microorganisms that confer health benefits on the host and have been extensively studied for their capacity to modulate gut microbiota, enhance intestinal barrier function, and attenuate inflammatory responses [27,28]. However, the use of live probiotics in vulnerable populations—such as the elderly, infants, or immunocompromised individuals—raises safety concerns [28,29]. In this context, the concept of postbiotics has emerged as a promising alternative. Postbiotics are defined as preparations of inanimate microorganisms and/or their metabolites that confer health benefits to the host [30]. Compared with live probiotics, postbiotics offer several advantages, including a well-defined chemical composition, extended shelf life, and, most importantly, a superior safety profile for sensitive populations such as the elderly, young children, and immunocompromised individuals [31].

The fermentation product used in this study, postbiotic Nagqu4580, is a freeze-dried powder derived from the mixed fermentation of multiple probiotic strains, including Streptococcus thermophilus S709, Lacticaseibacillus paracasei L578, and Lacticaseibacillus helveticus L551, followed by high-temperature and high-pressure sterilization. This process inactivates the bacterial cells while preserving their metabolites, such as gamma-aminobutyric acid (GABA), 5-hydroxytryptamine (5-HT), acetic acid, and malic acid. Postbiotic Nagqu4580 exhibits excellent acid resistance and contains a variety of bioactive components [5,6]. Given its composition of heat-inactivated bacterial cells and bioactive metabolites, Nagqu4580 qualifies as a postbiotic [32]. Notably, GABA and 5-HT, both of which are present in postbiotic Nagqu4580, have been shown to modulate gut motility, immune responses, and even host mood via the gut–brain axis [33,34,35,36]. These properties suggest that postbiotic Nagqu4580 may exert protective effects on intestinal health, making it a promising candidate for the management of UC.

To investigate the ameliorative effect of postbiotic Nagqu4580 on ferroptosis in inflamed epithelial cells in UC, the present study focuses on the following pathway. Postbiotic Nagqu4580 modulates the gut microbiota, which reshapes tryptophan metabolism by increasing beneficial metabolites such as *N*-acetyltryptophan (NAS) and 5-hydroxyindoleacetic acid (5-HIAA) while decreasing harmful metabolites such as 3-indoxyl sulfate (3-IS). This in turn activates intestinal AhR and Nrf2 signaling, inhibits intestinal epithelial ferroptosis, improves barrier function, and ultimately alleviates UC. This delineates its mechanism of action at the organismal level.

## 2. Materials and Methods

### 2.1. Preparation of Postbiotic Nagqu4580

The culture medium is mixed with 10–30 g/L of sodium glutamate, stirred at 55–60 °C for 10–15 min, and then pasteurized at 105–115 °C for 15–20 min. Subsequently, it is fermented with the probiotic, which is composed of 2–5% Streptococcus thermophilus S709, 10–20% Lactobacillus paracasei L578, and 75–88% Lactobacillus helveticus L551, at 37–45 °C for 28–35 h. When the pH reached 4.4–4.6, the mixture was stirred at 100–300 rpm, followed by sterilization treatment, and then vacuum freeze-dried to obtain Nagqu4580 powder. Lot/batch information, QC results, and inactivation confirmation of the prepared Nagqu4580 batch data is provided in the Supplementary Materials. The Nagqu4580 powder was provided by Shanghai Nature’s Sunshine Health Products Co., Ltd.

### 2.2. Combined Analysis of the Material Composition in Postbiotic Nagqu4580 by LC-MS/MS Metabolomics

Untargeted metabolomic analysis was performed using liquid chromatography–tandem mass spectrometry (LC-MS/MS). Sample preparation: Approximately 100 mg of postbiotic Nagqu4580 powder was weighed, mixed with 1 mL of methanol, vortexed for 10 min, and centrifuged at 12,000 rpm at 4 °C for 10 min. The supernatant was collected for injection. Chromatographic separation was carried out on a Thermo U3000 system (Thermo Fisher Scientific, Waltham, MA, USA) equipped with an ACQUITY Premier HSS T3 column (Waters Corporation, Milford, MA, USA).(2.1 × 100 mm, 1.8 μm) maintained at 45 °C. The mobile phase consisted of 0.1% formic acid in water (A) and methanol (B), with a flow rate of 0.3 mL/min. The gradient elution program was as follows: 0–1 min, 2% B; 1–5.5 min, increase to 100% B; maintain until 14 min; 14.1 min, decrease to 2% B; and re-equilibrate until 16 min. Mass spectrometric detection was conducted on a Q Exactive™ instrument operated in data-dependent acquisition (DDA) mode under both positive and negative ionization. Full-scan MS spectra were acquired at a resolution of 70,000 over the *m*/*z* range of 150–1500. The top 10 most intense precursor ions were selected for fragmentation with a resolution of 17,500, using stepped collision energies of 10, 30, and 55 eV. Raw data were processed using MS-DIAL software (version 4.70) for peak extraction, noise removal, deconvolution, and alignment, resulting in a three-dimensional data matrix containing retention time, *m*/*z*, and peak area. Metabolite identification was performed by matching the extracted MS features against public databases, including MassBank, ReSpect, and GNPS. The matching tolerances were set as follows: MS1 tolerance, 0.01 Da; MS2 tolerance, 0.05 Da; retention time tolerance, 0.05 min; and identification score cutoff > 80.

### 2.3. Animals

A total of 40 male C57BL/6 mice, aged 6–8 weeks and weighing 20–22 g, were included in this experiment. The mice utilized in this study were procured from Sibeifu Beijing Biotechnology Co., Ltd. (Beijing, China) and verified to be of specific pathogen-free (SPF) status, exhibiting robust physical health and no underlying diseases prior to the commencement of the experiment. All animals were maintained in a standardized barrier animal facility with controlled environmental conditions, including an ambient temperature of 22 ± 2 °C, relative humidity of 50–60%, and a 12 h light-dark cycle. The mice were housed in ventilated cages, with a maximum of five animals per cage, and were provided with ad libitum access to food and water. Following a 7-day acclimatization period, the formal experimental procedures were initiated. The animal study protocol was approved by the Ethics Committee of Xizang Minzu University (Ethics Approval No.: 2024-101).

### 2.4. Animal Grouping and Acute UC Modeling

Forty mice were randomly divided into 5 groups according to the random number table method, with 8 mice in each group: control group (NC), DSS group (UC), positive drug 5-ASA group (PC), postbiotic Nagqu4580 low-dose group (UC + NL), and postbiotic Nagqu4580 high-dose group (UC + NH). The sample size of 8 mice per group was determined using a power analysis based on preliminary pilot study data (α = 0.05, β = 0.20, power = 80%), accounting for an anticipated attrition rate of up to 25% during the DSS-induced acute phase to ensure sufficient statistical power for primary outcome analysis. During the experimental period, for the first 10 days, the NC, UC, and PC groups were administered normal water by gavage. Normal saline (0.9% sodium chloride solution) was used as the solvent for the bacterial powder to prepare postbiotic Nagqu4580. According to the “Methods for Evaluation of Health Food Function (2023 Edition)”, the dosages for experimental mice were set as low dose (1 g/kg) and high dose (3 g/kg), corresponding to 10 and 30 times the human dose, respectively. The NL and NH groups were administered postbiotic Nagqu4580 by gavage at doses of 1 g/kg and 3 g/kg, respectively. From day 4 onwards, except for the NC group, drinking water was supplemented with 4% (*w*/*v*) dextran sulfate sodium (DSS) solution to induce UC. All experimental procedures strictly adhered to the “3R” principles (Replacement, Reduction, Refinement): (1) Replacement: No non-animal alternatives were available for this in vivo modeling and whole-system efficacy assessment. (2) Reduction: The sample size was minimized through rigorous statistical planning while ensuring test validity. (3) Refinement: All manipulations, including intragastric administration and DSS drinking water replacement, were performed gently and quickly in a quiet environment to reduce unnecessary stress stimulation. Strict predefined humane endpoints were established in advance to avoid excessive animal suffering: (1) continuous body weight loss exceeding 20% of the initial body weight; (2) severe persistent bloody diarrhea, accompanied by obvious lethargy, anorexia and dyskinesia; and (3) extreme mental depression and inability to eat and drink independently. Any animal reaching these endpoints was humanely euthanized immediately via cervical dislocation under deep anesthesia (5% isoflurane) and excluded from subsequent analysis. Meanwhile, the PC group was given 100 mg/kg 5-aminosalicylic acid by gavage, and the UC + NL and UC + NH groups continued gavage at the aforementioned concentrations. On day 17, the mice were euthanized by cervical dislocation. During the acute phase, the disease activity index (DAI) score (body weight, stool consistency, and fecal bleeding) was monitored and recorded daily for all groups of mice, and animal deaths in each group were recorded. At the end of the experiment, the complete colon of each mouse was collected, its length was measured, and photographs were taken. The primary outcome measures of this study were predefined as DAI score and final colon length, which serve as the core quantitative indicators for evaluating UC model severity and the therapeutic efficacy of Nagqu4580 intervention. The secondary outcome measures included histological damage degree, inflammatory factor levels, ferroptosis-related molecular expression, gut microbiota composition, and serum tryptophan metabolite profiles, which were used to explore the potential molecular mechanism of Nagqu4580 alleviating ulcerative colitis.

During the entire experimental period, no accidental death of mice occurred. Individual sample size differences in different detection indicators were caused by standardized sample attrition based on experimental requirements and sample quality control, rather than random loss or data screening. The detailed sample attrition reasons and final valid sample size for each endpoint are as follows: (1) Colon length measurement and DAI score assessment (*n* = 6 per group): Two mice in each group were excluded because their colon tissues were damaged during material collection and could not meet the morphological measurement standards, and their daily behavioral observation records were incomplete, which failed to meet the data inclusion requirements; (2) Gut microbiota sequencing analysis (*n* = 5 per group): In addition to the above excluded individuals, one more mouse per group was excluded due to insufficient fecal sample collection and unqualified DNA extraction quality, which could not support subsequent 16S rRNA sequencing analysis; and (3) Histopathological H&E staining (*n* = 6 per group): To ensure the consistency and representativeness of tissue section staining and microscopic observation, only 6 mice with the most stable modeling status and complete tissue morphology in each group were selected for subsequent histological detection.

### 2.5. Real-Time Fluorescence Quantitative PCR

Total RNA was isolated using the Total RNA Extraction Kit (Biosharp, Beijing, China)and subsequently converted into cDNA via the Prime Script RT Reagent Kit. RT-qPCR was performed using cDNA as the template, TB Green Premix Ex Taq II as the premix, and the primers shown in Table 1.

### 2.6. Hematoxylin–Eosin (H&E) Staining

The fixed tissue was dehydrated, embedded, sectioned, dewaxed, stained with hematoxylin (Sigma Aldrich, Shanghai, China) for 5–10 min, differentiated with 3 s of acid alcohol solution, placed in a weak alkaline blue solution for re-blue, and then stained with eosin (Bomei, Hefei, China) for 3 min. It was then graded alcohol dehydrated, transparent with a clarifying agent, and sealed with neutral resin. The images of the sections were captured using a microscopic imaging system (Motic, Xiamen, China). Histological evaluation was conducted in a blinded manner.

### 2.7. Enzyme-Linked Immunosorbent Assay (ELISA)

The content of TNF-ɑ, IL-1β, IL-6 and 4-HNE in colon tissues was detected strictly according to the instructions of the ELISA kit (ZCIBio, Shanghai, China). Lipid peroxide MDA in colon tissue and GSH and GSSG content in colon tissue were detected by using the reagent kit (Jiancheng, Nanjing, China), and the GSH/GSSG ratio was calculated.

### 2.8. Immunofluorescence Staining

After dewaxing and hydration of paraffin sections, antigen retrieval was performed to expose antigenic epitopes. Endogenous peroxidase activity was inhibited using 3% H_2_O_2_ (Xilong, Wuhan, China), and non-specific sites were blocked with BSA (Servicebio, Wuhan, China). The first round of immunofluorescence staining was carried out: Incubate the sections with the primary antibody at 4 °C overnight, and then add the corresponding HRP-labeled secondary antibody and detect using the TSA fluorescence signal amplification system (Servicebio, Wuhan, China). For the second round of staining, the sections were subjected to antigen retrieval again to dissociate the first-round primary and secondary antibody complexes. Then, the second primary antibody was added and the operation was the same as that for the first primary antibody. Subsequently, the corresponding secondary antibody was reacted at 37 °C. Finally, nuclei were stained with DAPI (Servicebio, Wuhan, China) as a counterstain and mounted using an anti-fade mounting medium to preserve fluorescence for microscopic observation.

### 2.9. Western Blot Analysis

Prepare the cell protein extract, separate it by SDS-PAGE electrophoresis, and then transfer it to a PVDF membrane. After blocking, the PVDF membrane was incubated overnight at 4 °C with primary antibodies (GPX4, 1:15,000, ACSL4,1:2000, AhR, 1:2000, Nrf2, 1:2000 and anti-β-actin, 1:50,000) (Abclonal, Wuhan, China) (Huabio, Hangzhou, China) (Proteintech, Wuhan, China) (Affinity, Hangzhou, China). The membranes were washed with PBS three times and incubated with goat anti-Mouse IgG (H + L) secondary antibody (1:8000) (Abclonal, Wuhan, China) for 1 h at room temperature. The detection was performed using Torchlight’s Hypersensitive ECL substrate (Biosharp, Shanghai, China), and the imaging was carried out with the Tanon Fluorescence Image Analysis System (V2.0).

### 2.10. Metabolomic Analysis of Tryptophan and Its Metabolites in Serum

Serum samples were thawed on ice. Then, 50 μL of serum was mixed with chilled methanol containing internal standards for protein precipitation. After being vortex-mixed, the mixture was incubated on ice and centrifuged at 4 °C. The supernatant was collected and centrifuged again. Subsequently, 100 μL of the supernatant was transferred into an injection vial and temporarily stored at −20 °C until LC-MS/MS analysis. The data acquisition was performed using an ultra-performance liquid chromatography (UPLC) system (ExionLC™ AD, SCIEX, Framingham, MA, USA) coupled with a tandem mass spectrometry (MS/MS) system (QTRAP^®^ 6500+, SCIEX, Framingham, MA, USA). Metabolites were separated on a C18 column with a gradient elution using water and acetonitrile as the mobile phases. Mass spectrometry detection was conducted in both positive and negative electrospray ionization (ESI) modes with collision energy (CE) scanning. Strict quality control (QC) measures were implemented to ensure data reliability: pooled QC samples were prepared by mixing equal volumes of all serum samples, inserting every 10 samples during detection to monitor instrument stability and repeatability. The acquired mass spectrometry data were processed using Analyst 1.6.3 and MultiQuant 3.0.3 software. Standard calibration curves with 8 concentration gradient points were established for each target metabolite, with good linearity (R^2^ > 0.995) for quantitative calculation. The concentrations of metabolites were calculated based on standard curves, followed by normalization and subsequent statistical analysis. Metabolite identification was confirmed by matching retention time, parent ion mass-to-charge ratio, and characteristic fragment ions with authentic standard references.

### 2.11. 16S rRNA Gene-Based Gut Microbiota Analysis

Animal feces were collected, and microbial genomic DNA was extracted. After quality assessment by agarose gel electrophoresis and Nanodrop, the bacterial 16S rRNA gene V3-V4 region was amplified using primers 338F and 806R. The PCR products were purified using magnetic beads, quantified, and then used for library construction, followed by another round of purification and quality control. Finally, paired-end sequencing (2 × 250 bp) was performed on the Illumina NovaSeq 6000 platform. Strict sequencing quality control and standard bioinformatics processes were implemented: the minimum sequencing depth per sample was set to 50,000 clean reads to ensure sufficient microbial coverage. All raw sequencing reads were quality-filtered, trimmed, and normalized to uniform read counts for inter-sample comparison. Rarefaction curve analysis was performed to verify the adequacy of sequencing depth and community saturation. Operational taxonomic units (OTUs) were clustered at 97% sequence similarity, and taxonomic annotation was performed against the Silva 138 database. For beta-diversity analysis, Bray–Curtis and weighted UniFrac distance matrices were calculated, and principal coordinate analysis (PCoA) was performed to evaluate microbial community structural differences among groups. Statistical significance of beta-diversity differences was determined by permutational multivariate analysis of variance (PERMANOVA) with 999 permutations. False Discovery Rate (FDR) correction was applied for all multiple comparative analyses of microbial taxa and diversity indices to eliminate false positive results. All raw 16S rRNA sequencing data have been uploaded to the NCBI Sequence Read Archive (SRA) public repository, with the accession number: PRJNA1474718. Subsequent analyses were completed at Shanghai Biotree Biomedical Technology Co., Ltd.

### 2.12. Statistical Analysis

Data analysis was conducted with SPSS 20.0 and GraphPad Prism 8. Measurement data that passed normality tests are presented as mean ± standard deviation (Mean ± SD). Comparisons of means among multiple groups were performed using one-way analysis of variance (One-Way ANOVA). For all multiple pairwise comparisons after ANOVA, the false discovery rate (FDR) correction was strictly performed. If homogeneity of variance was confirmed, post hoc pairwise comparisons were conducted using the LSD test; if homogeneity of variance was not satisfied, Tamhane’s T2 test was applied instead. A *p* value < 0.05 was considered statistically significant.

## 3. Results

### 3.1. Identification of Postbiotic Nagqu4580 by LC-MS/MS Metabolomics

Untargeted LC-MS/MS analysis was employed to profile the chemical constituents of Nagqu4580 powder. A total of 498 substances were detected. Among these, 12 major compounds were identified with high confidence (overall identification score = 100) based on exact mass, isotopic pattern, and MS/MS spectral matching against reference databases (e.g., HMDB, MassBank). The detected compounds span diverse chemical classes, including amino acid derivatives, flavonoids, steroids, fatty acids/amides, and phospholipids, indicating the complex composition of the sample.

Key identifications (Supplementary Table S1) featured several bioactive natural products. Notably, salvianolic acid B, a renowned phenolic acid with cardiovascular protective effects, and isosakuranin, a flavonoid with antioxidant properties, were detected, suggesting potential plant-derived bioactivity in the sample. Additionally, mometasone furoate (a synthetic glucocorticoid) and methohexital (a barbiturate anesthetic) were identified. Considering that these synthetic pharmaceutical compounds are not inherent components of a postbiotic Nagqu4580, we speculate that these signals may come from extraneous interfering substances rather than intrinsic ingredients of the sample. The identification of lipids such as phosphatidylinositol (16:1–18:2) and stearamide reflects the sample’s lipidomic profile.

**Figure 1:**
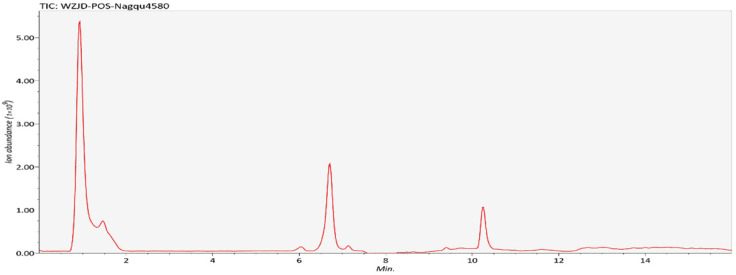
Total ion chromatogram of postbiotic Nagqu4580 in positive ion mode.

**Figure 2:**
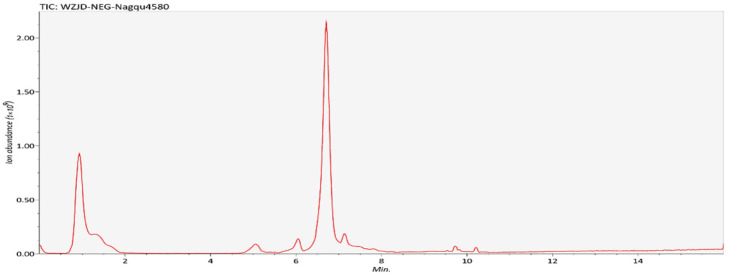
Total ion chromatogram of postbiotic Nagqu4580 in negative ion mode. Note: The *x*-axis represents retention time, and the *y*-axis represents ion intensity.

During chromatographic separation, the eluted components continuously enter the mass spectrometer, which performs continuous scanning for data acquisition. Each scan generates a mass spectrum, and the ion intensities at all time points from each mass spectrum are summed to obtain a total ion current intensity. A plot with ion intensity on the *y*-axis and time on the *x*-axis is then generated as the Total Ion Chromatogram (TIC). The TIC shown here was generated from one representative sample (Figure 1 and Figure 2).

### 3.2. Postbiotic Nagqu4580 Ameliorates Intestinal Inflammation and Restores Barrier Function in Mice with UC

**Figure 3:**
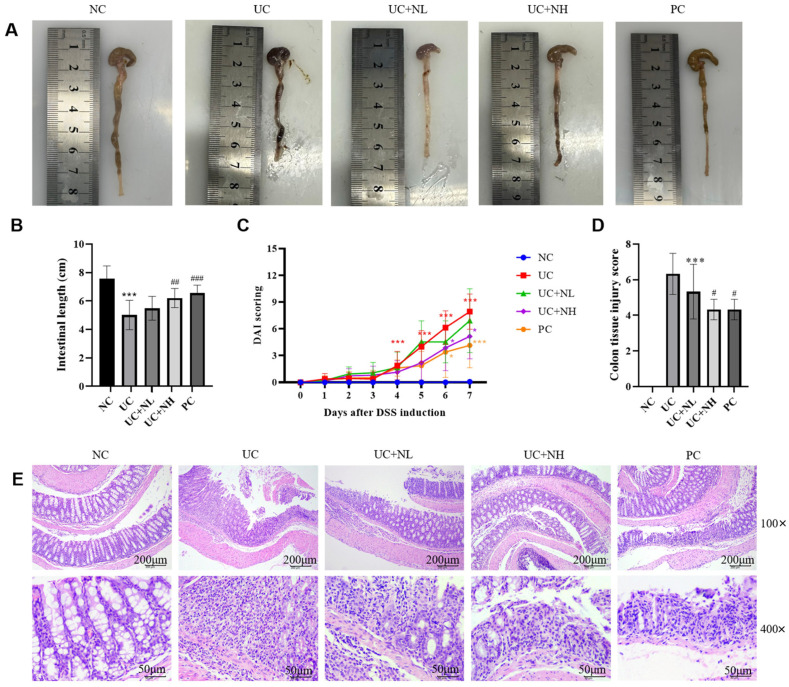
Postbiotic Nagqu4580 alleviates DSS-induced UC and protects colonic integrity. (**A**) Representative colon images; (**B**) the quantitative analysis of colon length (n = 6); (**C**) the DAI scores of each group during the acute phase of DSS induction (n = 6); (**D**,**E**) HE staining was used to observe the morphological changes of colon tissue and the pathological inflammation score (n = 6); Tamhane’s T2 test was applied for datasets in Figure 3D. Compared with the NC group, *** *p* < 0.001; compared with the UC group, ^#^ *p* < 0.05, ^##^ *p* < 0.01, ^###^ *p* < 0.001.

As shown in Figure 3A,B, compared with the NC, the colon length of mice in the UC was significantly shortened (*p* < 0.001). After intervention with different doses of the drug, the colon lengths of the UC + NL, UC + NH, and PC were all significantly restored compared with the UC group. Among them, the recovery effects of the UC + NH and PC groups were more significant (*p* < 0.001), and the UC + NL group also showed a marked improvement (*p* < 0.01). As shown in Figure 3C, the DAI score of the NC group remained at 0 throughout the entire experiment and no signs of disease were observed. The DAI score of the UC group continued to rise as the model construction time increased, reaching its peak on the 7th day. After drug intervention, the DAI scores of the UC + NH and PC groups were significantly lower than those of the UC group, while the UC + NL group showed a decreasing trend but was not significant. Additionally, the improvement effect of UC + NH was better than that of UC + NL and was comparable to that of the PC group. During the experiment, no death cases occurred in any group.

To systematically evaluate the interventional effect of postbiotic Nagqu4580 on UC in mice, we conducted a comprehensive analysis through histopathology, inflammatory factors, and intestinal barrier protein expression. The results indicate that postbiotic Nagqu4580 can dose-dependently ameliorate UC-induced colonic tissue damage, inflammatory response, and barrier dysfunction. HE staining revealed that, compared with the NC group, the colonic tissue damage score in the UC group was significantly higher (*p* < 0.001). After intervention with postbiotic Nagqu4580, the damage score in the low-dose group decreased, but the difference was not significant (*p* > 0.05), while both the high-dose group and the PC group significantly reduced the score (*p* < 0.05), suggesting that postbiotic Nagqu4580 at a high dose has a clear improving effect on colonic histopathology (Figure 3D,E).

**Figure 4:**
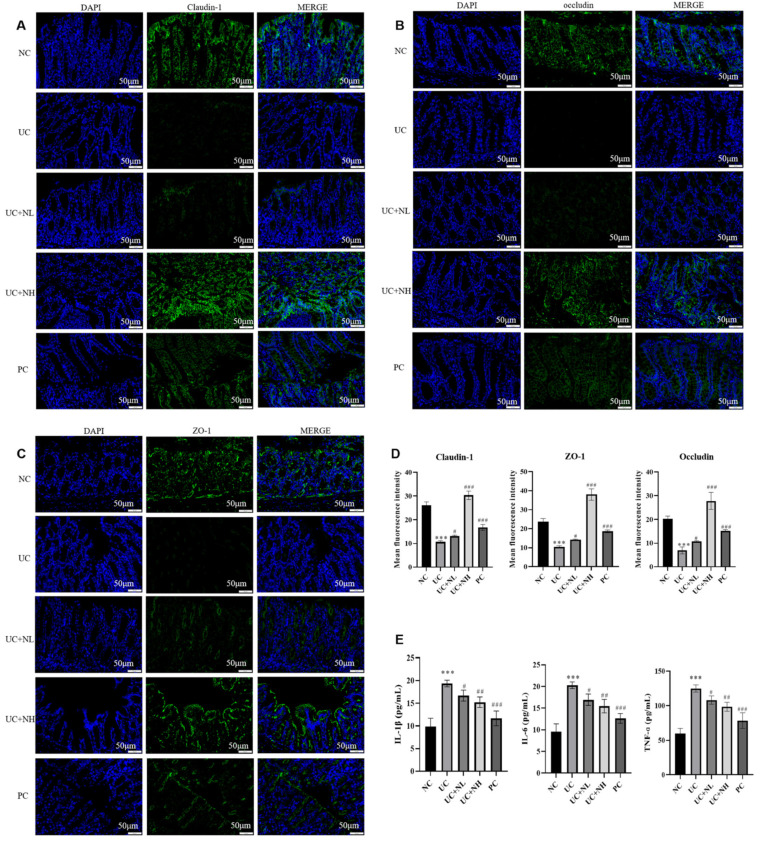
Postbiotic Nagqu4580 ameliorates intestinal inflammation and restores barrier function in mice with UC. (**A**–**D**) Immunofluorescence staining was used to observe the expressions of tight junction proteins ZO-1, occludin and Claudin-1 in the colon (n = 3); (**E**) ELISA was used to detect the contents of inflammatory cytokines TNF-α, IL-1β and IL-6 in colon tissue (n = 3). Compared with the NC group, *** *p* < 0.001; compared with the UC group, ^#^ *p* < 0.05, ^##^ *p* < 0.01, ^###^ *p* < 0.001.

Immunofluorescence staining further revealed that the expression of tight junction proteins Claudin-1, ZO-1, and Occludin in the colon of the UC group was significantly lower than that of the NC group (*p* < 0.001). The UC + NL group significantly increased the expression of all three proteins (*p* < 0.05), while the UC + NH group markedly upregulated their expression (*p* < 0.001), with effects comparable to those of the PC group, indicating that postbiotic Nagqu4580 has a significant restorative effect on intestinal barrier function in UC mice. ELISA detection of inflammatory factors showed that the levels of TNF-α, IL-1β, and IL-6 in the colon of the UC group were significantly elevated compared with the NC group (*p* < 0.001). All treatment groups reduced the levels of these inflammatory factors, with the low-dose group showing a significant effect (*p* < 0.05), the UC + NH group demonstrating a more pronounced reduction (*p* < 0.01), and the PC group achieving the most significant reduction (*p* < 0.001), indicating that postbiotic Nagqu4580 effectively inhibits UC-related inflammatory responses. In conclusion, postbiotic Nagqu4580, particularly at a high dose, can significantly alleviate colonic tissue damage, reduce inflammatory cytokine levels, and enhance the expression of tight junction proteins, thereby effectively mitigating intestinal inflammation and restoring barrier function (Figure 4).

### 3.3. Postbiotic Nagqu4580 Ameliorates Ferroptosis in Intestinal Epithelial Cells of UC Mice

**Figure 5:**
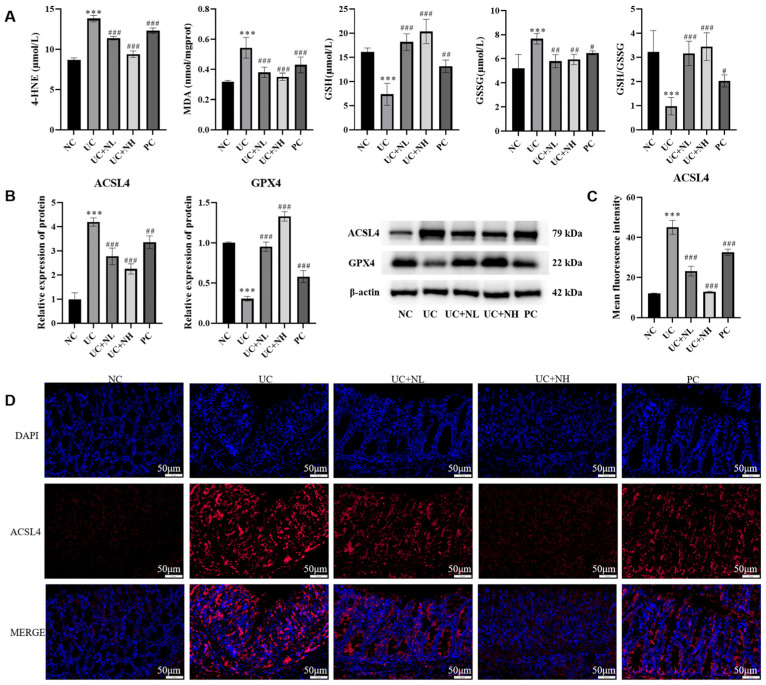
Postbiotic Nagqu4580 Ameliorates Ferroptosis in Intestinal Epithelial Cells of UC Mice (n = 3). (**A**) The kit was used to detect the lipid peroxides MDA and 4-HNE, as well as the contents of GSH and GSSG in colon tissue, and the ratio of GSH/GSSG was calculated; (**B**) WB was used to detect the expression of ferroptosis-related marker proteins GPX4 and ACSL4 in colon tissue; (**C**,**D**) immunofluorescence staining was used to observe the expression of ferroptosis-related marker protein ACSL4 in colonic epithelial cells. Compared with the NC group, *** *p* < 0.001; compared with the UC group, ^#^ *p* < 0.05, ^##^ *p* < 0.01, ^###^ *p* < 0.001.

This study evaluated the effect of postbiotic Nagqu4580 on ferroptosis in intestinal epithelial cells of UC mice by detecting the levels of lipid peroxidation in colonic tissues and the System Xc^−^/GSH/GPX4 antioxidant pathway. The results showed that the levels of lipid peroxidation products MDA and 4-HNE in the colon of the UC group were significantly higher than those in the NC group (*p* < 0.001), while after intervention with UC + NL, UC + NH or PC group, the levels of both were significantly reduced. Meanwhile, the content of GSH and the GSH/GSSG ratio in the colon of UC mice were significantly decreased, and the content of GSSG was increased (*p* < 0.001); each treatment group could reverse these changes to varying degrees and restore the balance of the antioxidant system (Figure 5A). Western blot and immunofluorescence staining further indicated that the expression of the ferroptosis-promoting protein ACSL4 in the colon tissue of the UC group was significantly upregulated (*p* < 0.001), while the expression of the key antioxidant protein GPX4 was significantly downregulated (*p* < 0.001); postbiotic Nagqu4580 intervention could dose-dependently reduce the expression of ACSL4 and increase the expression of GPX4 (*p* < 0.001), and its effect was comparable to or better than that of the PC group (Figure 5B–D). In conclusion, postbiotic Nagqu4580 can effectively inhibit the ferroptosis process in intestinal epithelial cells of UC mice by reducing lipid peroxidation, enhancing antioxidant defense, and regulating the expression of ACSL4/GPX4 proteins.

### 3.4. The Effect of Postbiotic Nagqu4580 on Tryptophan Metabolism in UC Mice

**Figure 6:**
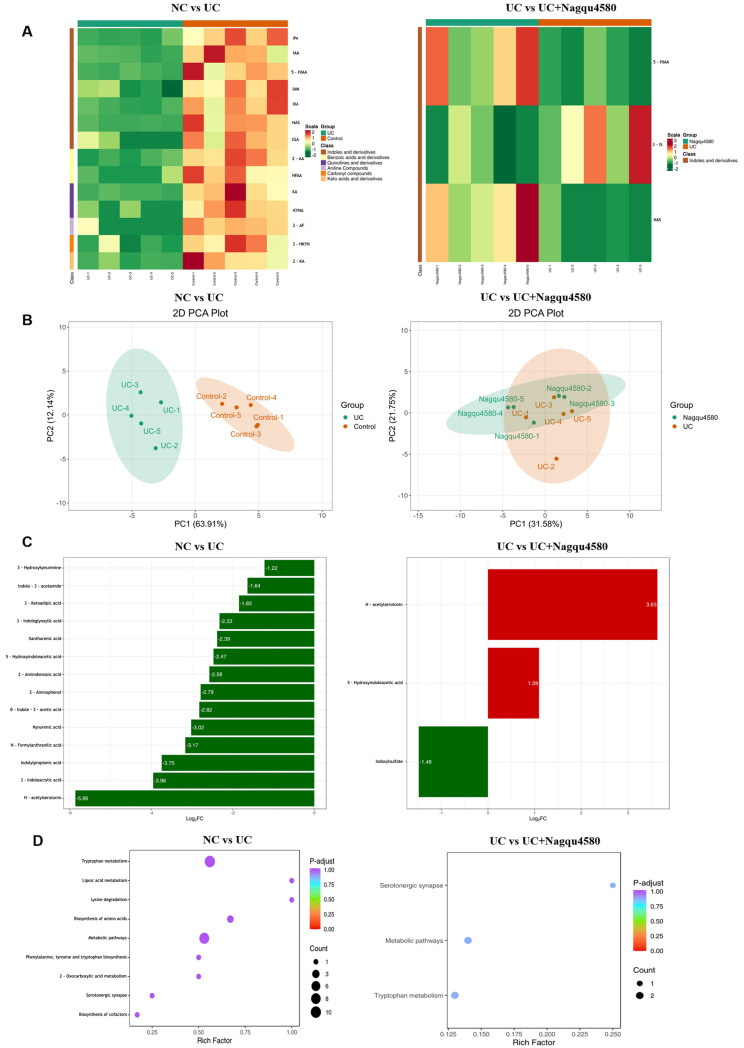
Metabolomics analysis of tryptophan and its metabolites in serum (n = 5). (**A**) Heatmaps; (**B**) PCA; (**C**) Top Fc Bar Metabolites; (**D**) KEGG.

Unsupervised hierarchical clustering analysis was performed to visualize the global metabolic differences between groups (Figure 6A). In the NC vs. UC comparison, a clear separation was observed between the two groups, with distinct color gradients indicating widespread metabolic perturbations in UC mice relative to NC controls. In the UC vs. UC + postbiotic Nagqu4580 comparison, the heatmap revealed a distinct clustering pattern between UC and postbiotic Nagqu4580-treated groups. The color distribution indicated that postbiotic Nagqu4580 treatment partially reversed the metabolic dysregulation observed in UC mice, with a notable shift toward the metabolic profile of healthy controls.

Principal component analysis (PCA) was conducted to assess the overall separation between groups (Figure 6B). In the NC vs. UC plot, the two groups formed distinct, non-overlapping clusters along the first principal component, confirming a clear metabolic distinction between healthy and colitic states. The second principal component further separated individual samples within each group, reflecting inter-individual variability. In the UC vs. UC + postbiotic Nagqu4580 plot, PCA also demonstrated a clear separation between UC and treated groups, indicating that postbiotic Nagqu4580 treatment induced a significant metabolic shift in UC mice. The treated samples formed a distinct cluster, suggesting a consistent therapeutic effect on the metabolic phenotype of colitis.

Metabolite analysis identified significantly altered metabolites between groups (Figure 6C). In UC vs. NC, a total of 14 metabolites were significantly dysregulated, with the majority being downregulated in UC mice. Key downregulated metabolites included NAS, 3-indoleacrylic acid (IA), and Indolylpropionic acid, indicating profound perturbations in tryptophan metabolism and amino acid catabolism in colitis. In UC + postbiotic Nagqu4580 vs. UC, three metabolites were significantly altered. Notably, NAS and 5-HIAA were strongly upregulated, while Indoxylsulfate was downregulated, suggesting that postbiotic Nagqu4580 treatment specifically modulates tryptophan and indole derivative metabolism to alleviate colitic symptoms.

Metabolic pathway enrichment analysis was performed to identify the biological processes underlying the observed metabolic changes (Figure 6D). In NC vs. UC, the top enriched pathways included tryptophan metabolism, lysine degradation, and biosynthesis of amino acids, highlighting the central role of amino acid metabolic reprogramming in the pathogenesis of colitis. In UC vs. UC + postbiotic Nagqu4580, the most significantly enriched pathways were serotonergic synapse and tryptophan metabolism, indicating that postbiotic Nagqu4580 exerts its therapeutic effects, at least in part, by regulating tryptophan metabolism and serotonergic signaling pathways, which are closely linked to intestinal inflammation and barrier function.

**Figure 7:**
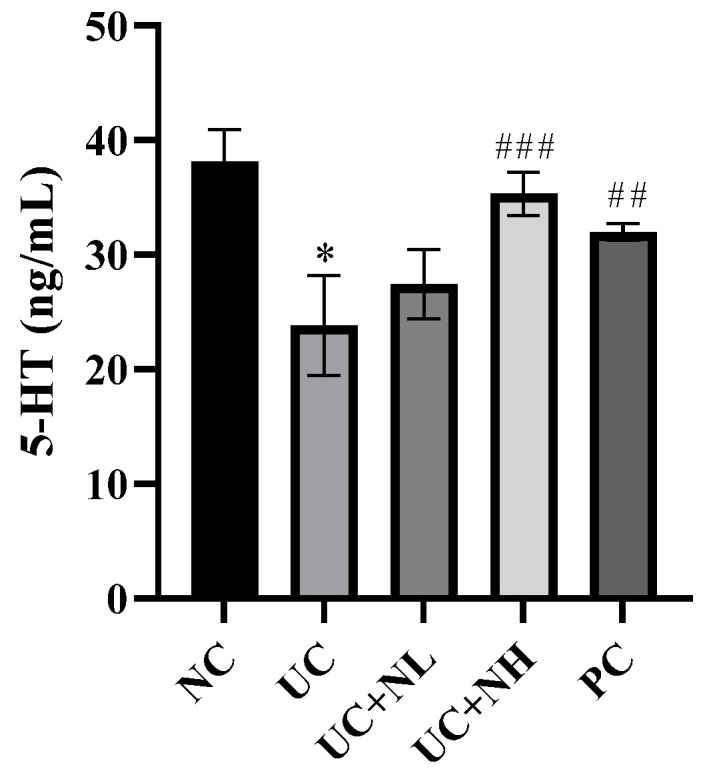
ELISA detection of 5-HT content in colon tissue (n = 6). Note: Compared with the NC group, * *p* < 0.05; compared with the UC group, ^##^ *p* < 0.01, ^###^ *p* < 0.001.

ELISA detection of 5-HT content in colon tissue (Figure 7): The results showed that, compared with the NC group, the level of 5-HT in the colon of the UC group was significantly decreased (*p* < 0.001). Compared with the UC group, no statistically significant difference was observed in the UC + NL group (*p* > 0.05), whereas the 5-HT level was significantly increased in the UC + NH group (*p* < 0.001) and the PC group (*p* < 0.01).

### 3.5. Postbiotic Nagqu4580 Modulates Gut Microbiota Dysbiosis in Species Diversity Analysis of UC Mice

**Figure 8:**
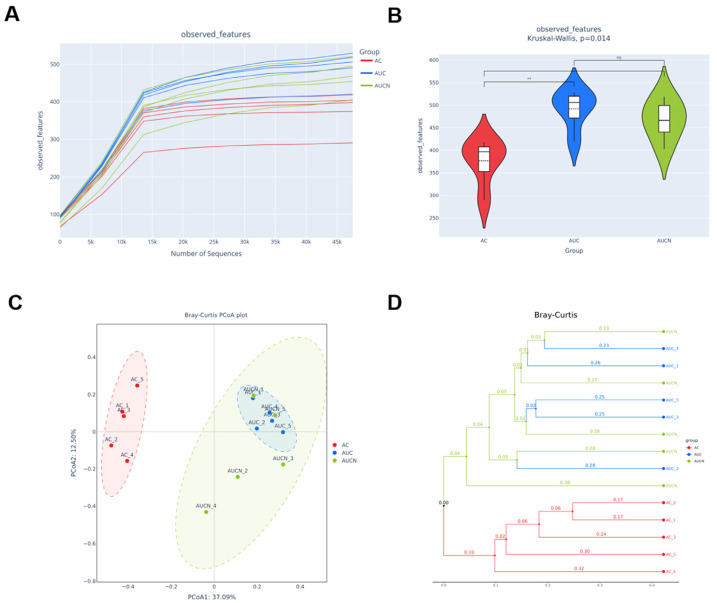
Species Diversity Analysis (n = 5). (**A**) Dilution Curve; (**B**) Violin Plot; (**C**) PCoA Plot; (**D**) UPGMA Clustering Tree Diagram.AC: NC group; AUC: UC group; AUCN: UC + NH group. Note: In panels (**B**): ** indicates *p* < 0.01, ns indicates not significant (*p* ≥ 0.05), and the dotted line represents the median value.

To investigate the effect of postbiotic Nagqu4580 on gut microbiota in UC mice, 16S rRNA gene sequencing was performed on fecal samples (n = 15, 5 per group). Rarefaction curves plateaued, indicating sufficient sequencing depth (Figure 8A). Alpha diversity assessed by Observed_features showed a significant increase in the UC group vs. NC (*p* = 0.014, Kruskal–Wallis), which was partially reduced by high-dose postbiotic Nagqu4580 (*p* > 0.05 vs. UC) (Figure 8B). Beta diversity analysis using PCoA (Bray–Curtis) revealed distinct clustering of NC and UC groups along PC2 (37.09% and 12.50% variance explained). Notably, mice treated with postbiotic Nagqu4580 showed a scattered distribution: some individuals (e.g., AUCN_4) clustered closer to the NC group, while others (AUCN_2, AUCN_3) were positioned between the control and UC groups. This pattern suggests that postbiotic Nagqu4580 treatment partially reversed the DSS-induced dysbiosis, albeit with inter-individual variation (Figure 8C). UPGMA clustering further showed that NC samples formed a distinct branch, UC samples were dispersed, and most postbiotic Nagqu4580-treated samples clustered intermediately, indicating partial restoration of gut microbiota composition toward healthy controls (Figure 8D).

### 3.6. Postbiotic Nagqu4580 Restores Gut Microbiota Homeostasis in UC as Revealed by Species Composition and Difference Analyses

**Figure 9:**
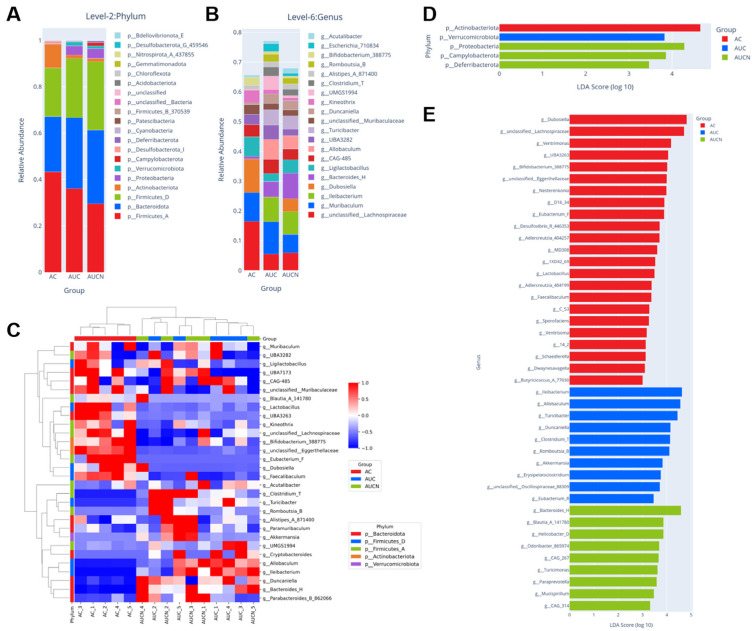
Species composition analysis and analysis of species differences (n = 5). (**A**,**B**) Taxonomic Composition Bar Chart (Phylum/Genus); (**C**) Species Composition Heatmap (Genus); (**D**,**E**) Bar Chart of LEfSe LDA Effect Values. AC: NC group; AUC: DSS group; AUCN: postbiotic Nagqu4580 high-dose group.

At the phylum level, the dominant phyla in all samples were Firmicutes and Bacteroidota, with their combined relative abundance exceeding 85%. Compared with the NC group, the relative abundance of Proteobacteria was significantly increased in the UC group (*p* < 0.01), while the abundance of Actinobacteria was decreased. After postbiotic Nagqu4580 intervention, the abundance of Proteobacteria slightly increased, while the abundance of Actinobacteria remained essentially unchanged compared to the AUC group (Figure 9A).

The relative abundance of the intestinal microbiota at the genus level was analyzed among the three groups (Figure 9B). The results showed that the NC group had a stable intestinal microbiota structure, with Muribaculum, Dubosiella, Ligilactobacillus, and unclassified Lachnospiraceae as the core dominant genera, which is consistent with the intestinal microecological characteristics of healthy mice. Compared with the NC group, the UC group exhibited significant dysbiosis: the abundances of beneficial genera Dubosiella and Ligilactobacillus decreased, while the abundances of pro-inflammatory genera such as Ileibacterium and Allobaculum abnormally increased. Meanwhile, the abundance of unclassified Lachnospiraceae decreased, impairing microbial diversity and balance. After high-dose postbiotic Nagqu4580 intervention, the intestinal microbiota structure of UC mice was significantly improved: the abundances of beneficial bacteria such as Ligilactobacillus and Dubosiella markedly rebounded compared with the UC group, and the abundances of pro-inflammatory bacteria such as Ileibacterium and Allobaculum were restored toward normal levels. The microbial composition recovered toward that of the NC group, suggesting that high-dose postbiotic Nagqu4580 intervention can effectively reshape the disturbed intestinal microecology in UC mice.

To further clarify the abundance distribution patterns and intergroup differences of the intestinal microbiota at the genus level among the three groups, a clustering heatmap was constructed based on the genus-level relative abundance data (Figure 9C). The results showed clear within-group clustering and between-group separation: The fecal microbiota samples of the NC and UC groups formed completely distinct clustering branches, indicating that DSS modeling induced a significant remodeling effect on the overall structure of the gut microbiota. In contrast, samples from the high-dose postbiotic Nagqu4580 group were clearly separated from the UC group, with some samples exhibiting microbiota characteristics that approached those of the NC group, suggesting that postbiotic intervention could partially reverse DSS-induced dysbiosis and promote the restoration of a healthy microbiota homeostasis. Further analysis of genus-level abundance revealed that, compared with the NC group, the UC group exhibited significantly reduced abundances of multiple known beneficial commensal genera, including the short-chain fatty acid-producing Muribaculum and Faecalibaculum, as well as the anti-inflammatory Ligilactobacillus. In contrast, opportunistic pathogenic genera associated with intestinal inflammation and barrier damage, such as Turicibacter, Ileibacterium, and Duncaniella, were significantly enriched in the UC group. After high-dose postbiotic Nagqu4580 intervention, the abundances of the above-mentioned beneficial genera in the high-dose postbiotic Nagqu4580 group were slightly increased compared with the UC group, while the abundances of pro-inflammatory-associated genera were markedly reduced. Notably, the high-dose postbiotic Nagqu4580 group also exhibited some unique genus-level features distinct from the NC group, such as enrichment of Parabacteroides_B, suggesting that probiotic intervention does not simply restore a healthy microbiota but rather establishes a novel microbiota homeostasis with anti-inflammatory potential. Additionally, Akkermansia, belonging to the phylum Verrucomicrobiota, showed significantly increased abundance in the UC group, indicating that this genus may be closely associated with DSS-induced intestinal barrier damage and inflammatory status. Collectively, these results demonstrate that postbiotic Nagqu4580 can effectively ameliorate DSS-induced gut dysbiosis by targeted regulation of the abundances of key bacterial genera, providing direct microbiota-level evidence for their alleviating effects on colitis.

The LEfSe (Linear Discriminant Analysis) results showed that there were significant intergroup-specific enrichment characteristics in the gut microbiota of the three groups of mice (LDA score > 3.0, *p* < 0.05). At the phylum level (Figure 9D), Actinobacteriota was significantly enriched in the NC group, Verrucomicrobiota was specifically increased in the UC group, while Proteobacteria, Campylobacterota, and Deferribacterota were the characteristic enriched phyla in the postbiotic Nagqu4580 high-dose intervention group. At the genus level (Figure 9E), it was further shown that the NC group had beneficial genera such as Dubosiella, unclassified Lachnospiraceae, and Lactobacillus as the core enriched taxa; the UC group significantly enriched pro-inflammatory-related genera such as Ileibacterium, Allobaculum, and Akkermansia; and after postbiotic Nagqu4580 intervention, genera with anti-inflammatory and intestinal barrier protective functions such as Bacteroides_H, Blautia_A_141780, and Helicobacter_D were significantly upregulated, suggesting that Nagqu4580 can effectively reverse UC-induced intestinal microecological disorders and reshape gut microbiota homeostasis by targeting specific differential bacteria.

### 3.7. Postbiotic Nagqu4580 Activates the AhR-Nrf2 Signaling Pathway to Restore Gut Homeostasis in UC Mice

**Figure 10:**
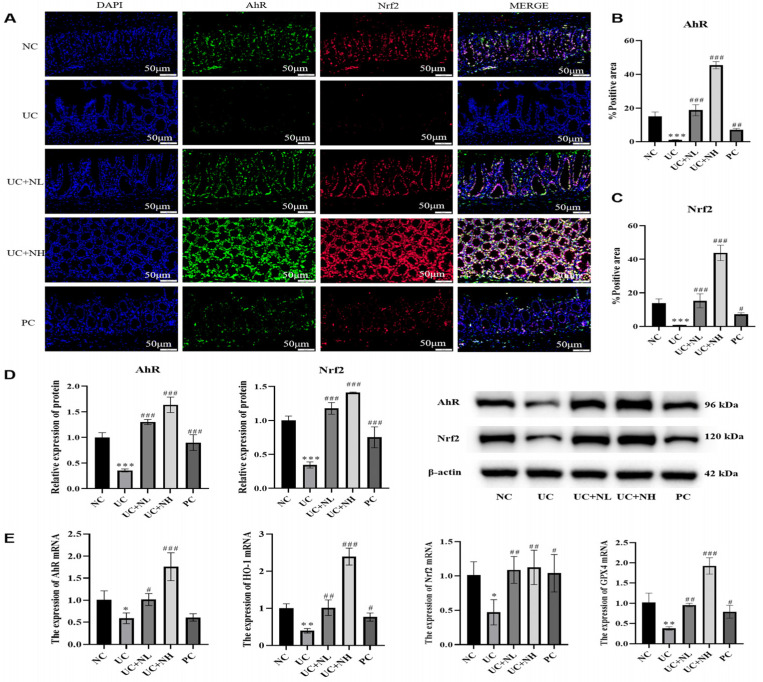
Regulation of the AhR-Nrf2 signaling pathway by postbiotic Nagqu4580 in UC mice (n = 3). (**A**–**C**) Double immunofluorescence staining was used to observe the co-expression of AhR and Nrf2 in colonic epithelial cells; (**D**) WB was used to detect AhR and Nrf2 in colon tissue; (**E**) the mRNA expressions of AhR, HO-1, Nrf2 and GPX4 were detected by qPCR. Compared with the NC group, * *p* < 0.05, ** *p* < 0.01, *** *p* < 0.001; compared with the UC group, ^#^ *p* < 0.05, ^##^ *p* < 0.01, ^###^ *p* < 0.001.

To investigate whether postbiotic Nagqu4580 exerts its effects via the AhR-Nrf2 signaling pathway, we systematically evaluated the pathway at the levels of protein localization, expression, and gene transcription (Figure 10). Immunofluorescence double staining showed that, compared with the NC group, the positive expression of both AhR and Nrf2 was significantly reduced in the colonic epithelial cells of UC mice (*p* < 0.001). After intervention with postbiotic Nagqu4580, the expression of AhR and Nrf2 increased in a dose-dependent manner (*p* < 0.001), and the 5-ASA group also significantly upregulated their expression (AhR *p* < 0.01; Nrf2 *p* < 0.05). Western blot results further confirmed this trend at the protein level: the expression of AhR and Nrf2 proteins was significantly downregulated in the colon tissue of the UC group (*p* < 0.001), while all treatment groups significantly restored their expression (*p* < 0.001). At the gene transcription level, RT-qPCR results showed that the mRNA expression of AhR, Nrf2, and their downstream target genes HO-1 and GPX4 was significantly decreased in the UC group. Postbiotic Nagqu4580 intervention effectively reversed the transcription levels of these genes, with the UC + NH group showing particularly significant upregulatory effects on all indicators (AhR, GPX4, HO-1 *p* < 0.001; Nrf2 *p* < 0.01) and demonstrating a clear dose–response relationship. The PC group also upregulated the mRNA expression of Nrf2, HO-1, and GPX4, but had no significant effect on AhR mRNA levels. In summary, postbiotic Nagqu4580 comprehensively activates the suppressed AhR-Nrf2 signaling pathway in the colon of UC mice at both transcriptional and protein levels, and upregulates the expression of downstream antioxidant genes.

## 4. Discussion

Ulcerative colitis is a chronic inflammatory bowel disease characterized by disrupted intestinal barrier function, dysregulated immune responses, and oxidative stress-induced epithelial damage. Recent advances have highlighted the complex interplay between programmed cell death pathways—particularly ferroptosis—in the pathogenesis of UC [37]. Emerging evidence has implicated ferroptosis, a non-apoptotic, iron-dependent form of cell death, in intestinal epithelial cell injury [13]. In this study, we demonstrated that postbiotic Nagqu4580, a probiotic fermentation product, significantly ameliorates DSS-induced acute colitis in mice by inhibiting intestinal epithelial ferroptosis through the microbiota–tryptophan–AhR/Nrf2 axis.

Our results showed that postbiotic Nagqu4580 treatment dose-dependently alleviated colonic inflammation, restored tight junction protein expression (Claudin-1, ZO-1, Occludin), and reduced inflammatory cytokine levels (TNF-α, IL-1β, IL-6). These findings are consistent with previous reports that targeting ferroptosis and oxidative stress can effectively mitigate UC symptoms. Notably, postbiotic Nagqu4580 suppressed lipid peroxidation markers (MDA, 4-HNE) and restored the GSH/GSSG balance, indicating a potent antioxidant effect. The downregulation of ACSL4 and upregulation of GPX4 further confirmed the inhibition of ferroptosis in colonic epithelial cells, providing direct evidence that the therapeutic effect of postbiotic Nagqu4580 is closely linked to the suppression of this specific cell death pathway [13].

Furthermore, our study found that postbiotic Nagqu4580 significantly improved DSS-induced gut microbiota dysbiosis, which aligns with current research trends in the field of postbiotics. Numerous studies have shown that modulating the gut microbiota through supplementation with specific probiotics, prebiotics, or postbiotics can effectively promote host health and alleviate intestinal inflammation. For example, a systematic review by Smolinska et al. [26] indicated that various probiotics, prebiotics, synbiotics, and postbiotics exert protective effects in multiple diseases, including IBD, by promoting the growth of beneficial microorganisms, enhancing the intestinal barrier, and modulating immune responses. Notably, Han et al. [38] reported that heat-inactivated Bifidobacterium M1-3 postbiotics alleviated DSS-induced colitis in mice by regulating the gut microbiota, promoting tryptophan metabolism, and activating the AhR/IL-22 signaling pathway. Chen et al. [39] also demonstrated that Lacticaseibacillus paracasei L21 and its heat-inactivated postbiotic preparation improved UC by modulating the gut microbiota, restoring the intestinal barrier, and activating the HIF1α/AhR-IL-22 axis. Collectively, these studies suggest that postbiotics, as a safe and effective alternative strategy, hold broad application prospects in the treatment of UC.

To further elucidate the molecular basis underlying the alleviation of UC by postbiotic Nagqu4580, this study analyzed its chemical composition using LC-MS/MS untargeted metabolomics. The results showed that postbiotic Nagqu4580 contains several bioactive metabolites with well-defined activities, including salvianolic acid B, isosakuranin, and linoleamide. Published studies have demonstrated that these components possess antioxidant, anti-inflammatory, or anti-lipid peroxidation properties, thereby providing a reliable material basis for the therapeutic effects of postbiotic Nagqu4580. Specifically, salvianolic acid B, a major water-soluble component of Salvia miltiorrhiza, has been shown in a DSS-induced mouse colitis model to reduce the disease activity index, alleviate mucosal damage and inflammatory cell infiltration, increase short-chain fatty acid production, and modulate gut microbiota composition, with its mechanism of action closely linked to its antioxidant activity [40]. Isosakuranin, a natural flavonoid, has been confirmed to exert antioxidant and anti-inflammatory activities, playing a positive role in experimental colitis by downregulating the NF-κB signaling pathway [41]. Linoleamide, an endocannabinoid-like fatty acid amide, inhibits the NF-κB signaling pathway in mouse RAW264.7 macrophages and exerts significant anti-inflammatory effects [42]. These metabolites exert anti-inflammatory and antioxidant functions through distinct molecular targets, and their synergistic presence in postbiotic Nagqu4580 provides a compositional explanation for the reliable material basis by which this postbiotic preparation inhibits intestinal epithelial ferroptosis and alleviates DSS-induced colitis.

A key finding of this study is the significant modulation of tryptophan metabolism by postbiotic Nagqu4580. Untargeted and targeted metabolomic analyses revealed that DSS-induced colitis caused a profound perturbation in tryptophan metabolism, which was partially reversed by postbiotic Nagqu4580 treatment. Specifically, postbiotic Nagqu4580 decreased the levels of potentially harmful metabolites like *N*-acetyltryptophan and increased the production of beneficial indole derivatives, such as indolebutyric acid and 5-HIAA. The increase in colonic 5-HT content further supports a shift towards the serotonin pathway. Tryptophan metabolites, particularly those derived from the gut microbiota, are known to be potent ligands for the aryl hydrocarbon receptor (AhR), a transcription factor that plays a critical role in intestinal immune regulation and barrier integrity [43]. The decrease in 3-indoxyl sulfate (3-IS), a microbial-derived uremic toxin, and the increase in 5-HIAA suggest that postbiotic Nagqu4580 may remodel the gut microbiota to favor a protective metabolic profile, redirecting tryptophan away from harmful degradation pathways and towards AhR-activating ligands.

To directly assess the impact of postbiotic Nagqu4580 on gut microbiota, we performed 16S rRNA gene sequencing of fecal samples. Our results demonstrated that postbiotic Nagqu4580 treatment partially reversed DSS-induced gut dysbiosis. Alpha diversity analysis showed that the increased observed features in UC mice were reduced by postbiotic Nagqu4580, though not to normal levels. Beta diversity (PCoA) revealed that treated samples scattered between control and UC groups, indicating a partial restoration of microbial community structure. This heterogeneity in response among postbiotic Nagqu4580-treated mice may be attributed to several factors, including individual variations in initial microbiota composition, differences in the extent of DSS-induced colitis severity, and potential variations in host physiological responses to the postbiotic intervention. Nevertheless, the overall shift toward the NC group suggests a modulatory effect of postbiotic Nagqu4580. At the phylum level, after postbiotic Nagqu4580 intervention, the abundance of Proteobacteria showed a slight increase, and the abundance of Actinobacteria remained essentially unchanged compared to the UC group. This elevation was mainly caused by non-pathogenic genera such as *Escherichia*, *Sphingomonas* and *Psychrobacter*, which are common commensals in the gut and do not induce intestinal inflammation. At the genus level, postbiotic Nagqu4580 significantly restored beneficial bacteria such as Dubosiella and Ligilactobacillus, while reducing pro-inflammatory genera, including Ileibacterium and Allobaculum.

LEfSe analysis further revealed statistically robust taxonomic signatures that distinguished the gut microbiota of control, UC, and postbiotic Nagqu4580-treated groups. In UC mice, pro-inflammatory-associated genera such as Ileibacterium, Allobaculum, and Akkermansia were significantly enriched, consistent with their reported roles in promoting intestinal inflammation. Notably, postbiotic Nagqu4580 intervention led to a marked enrichment of several genera with known anti-inflammatory or barrier-protective potential, including Bacteroides_H, Blautia_A_141780, and Helicobacter_D. Among these, Blautia species have been shown to produce short-chain fatty acids and indole derivatives, which can serve as AhR ligands. Bacteroides strains are also recognized for their tryptophan-metabolizing capacity, generating metabolites that modulate host immune responses. Thus, the LEfSe-identified shifts are not merely compositional but likely functional, directly linking postbiotic Nagqu4580-induced microbial remodeling to the production of AhR-activating tryptophan metabolites. These findings reinforce the concept that postbiotic Nagqu4580 rebalances the gut ecosystem by selectively promoting beneficial, metabolically active taxa, thereby suppressing ferroptosis via the microbiota–tryptophan–AhR/Nrf2 axis. Regrettably, the current study failed to perform quantitative correlation analysis between the relative abundance of these two key genera and the concentrations of intestinal protective tryptophan metabolites, which restricts the direct causal proof linking postbiotic Nagqu4580-mediated microbiota modulation to tryptophan-AhR signaling activation. Future in vitro and in vivo validation, including targeted metabolomics quantification and Spearman correlation calculation, will be carried out to explicitly confirm this microbiota–tryptophan metabolic axis, further clarifying the intermediate regulatory mechanism of postbiotic Nagqu4580.

The activation of the AhR pathway and its crosstalk with the oxidative stress response regulator Nrf2 is a central mechanism linking our observations. Our data demonstrate that postbiotic Nagqu4580 significantly upregulated AhR and Nrf2 expression at both the protein and mRNA levels in colonic tissues. This activation extended to their downstream targets, HO-1 and GPX4, which are critical for combating oxidative stress and lipid peroxidation. Critically, this points to an indirect, host-mediated antioxidant mechanism. The protective effect against oxidative damage is not primarily due to direct radical scavenging by metabolites present in the postbiotic, but rather the result of Nagqu4580 activating the AhR/Nrf2 axis, which in turn upregulates the host’s own enzymatic antioxidant defenses. The co-localization and increased expression of AhR and Nrf2 in colonic epithelial cells, as shown by immunofluorescence, further strengthens this mechanistic link. The intricate crosstalk between AhR and Nrf2 is increasingly recognized as a vital defensive axis in the gut–liver axis, where their combined activation helps maintain intestinal barrier function and mitigate inflammation [44]. These results align perfectly with a growing body of evidence showing that AhR activation can inhibit ferroptosis. For instance, studies have shown that AhR agonists like FICZ and ILA can protect against ferroptosis in models of kidney injury and stroke by upregulating the Nrf2/GPX4/SLC7A11 axis [22,23]. Our findings extend this concept to the context of UC, showing that a complex postbiotic like postbiotic Nagqu4580 can harness this pathway in vivo to protect the intestinal epithelium.

It is noteworthy that while 5-ASA, a first-line UC drug, also upregulated Nrf2 and its target genes, it did not significantly affect AhR mRNA levels. This distinction underscores the unique multimodal mechanism of postbiotic Nagqu4580, which appears to act upstream by modulating microbial metabolism to generate endogenous AhR ligands, thereby triggering a broader and more physiological activation of the AhR/Nrf2 cytoprotective axis. This is consistent with studies showing that the anti-ferroptotic effects of microbial metabolites like ILA are AhR-dependent [22,23,25,26,27,28,29,30,31,32,33,34,35,36,37,38,39,40,41,42,43,44,45]. The ability of postbiotic Nagqu4580 to both increase protective metabolites (like 5-HIAA, a potential AhR ligand) and decrease toxic ones (like 3-IS) highlights its potential to holistically restore metabolic homeostasis in the inflamed gut. Therefore, we propose that the core antioxidant mechanism of postbiotic Nagqu4580 is the transcriptional activation of endogenous cytoprotective enzymes via AhR/Nrf2 signaling, a strategy that offers a more sustained and systems-level protection compared to the finite and transient chemical antioxidant activity of its constituent metabolites.

In summary, this study provides evidence that postbiotic Nagqu4580 alleviates UC by inhibiting intestinal epithelial ferroptosis via the microbiota tryptophan AhR/Nrf2 axis. By reshaping tryptophan metabolism toward protective pathways (e.g., NAS and 5-HIAA) and reducing harmful metabolites such as 3-IS, postbiotic Nagqu4580 restores redox balance and barrier function in the inflamed colon. These findings offer new insights into the therapeutic potential of probiotic-derived postbiotics in IBD and support further development of postbiotic Nagqu4580 as a functional food or adjunct therapy for UC.

Despite these promising findings, this study has several limitations. First, while we observed changes in microbiota-related metabolites, direct metagenomic sequencing is needed to confirm the specific shifts in gut microbial composition and function induced by postbiotic Nagqu4580. Second, the causal role of specific tryptophan metabolites in mediating the anti-ferroptotic and AhR-activating effects requires validation through in vitro experiments or in vivo metabolite supplementation studies. Third, the use of conditional knockout mice would help establish the necessity of this pathway in mediating the therapeutic effects of postbiotic Nagqu4580. Notably, although postbiotic Nagqu4580 contains a variety of bioactive metabolites that jointly exert colitis-alleviating effects, the precise functional contribution and independent efficacy of each individual compound to the overall protective phenotype remain unclarified in the current study. It is still unclear which key metabolite dominates the regulation of gut microbiota homeostasis, tryptophan metabolism, and AhR-Nrf2 signaling activation, and the potential synergistic or antagonistic interactions among different metabolites also require further systematic exploration. Future studies should address these points to further elucidate the precise molecular mechanisms and evaluate the translational potential of postbiotic Nagqu4580 for UC treatment.

## 5. Conclusions

In conclusion, this study demonstrates that postbiotic Nagqu4580, a probiotic fermentation product, effectively alleviates DSS-induced ulcerative colitis in mice. The therapeutic mechanism is multifaceted, involving the inhibition of intestinal epithelial ferroptosis, restoration of the colonic antioxidant system, and improvement of barrier function. These effects are mediated, at least in part, by the modulation of tryptophan metabolism and the subsequent activation of the cytoprotective AhR/Nrf2 signaling axis. Our findings position postbiotic Nagqu4580 as a promising postbiotic candidate for the management of UC.

## Figures and Tables

**Table 1:** Primers sequence.

Gene	Primer Sequence-F	Primer Sequence-R
β-actin	CTACCTCATGAAGATCCTGACC	CACAGCTTCTCTTTGATGTCAC
AhR	CATCGACATAACGGACGAAATC	CTGTTGCTGTTGCTCTAGTTG
Nrf2	CAGCCATGACTGATTTAAGCAG	CAGCTGCTTGTTTTCGGTATTA
HO-1	TCCTTGTACCATATCTACACGG	GAGACGCTTTACATAGTGCTGT
GPX4	CCCGATATGCTGAGTGTGGTTTAC	TTTCTTGATTACTTCCTGGCTCCTG

## Data Availability

The original contributions of this study are documented in the article/Supplementary Materials. Further inquiries may be directed to the corresponding author.

## Supplementary Materials

The following supporting information can be downloaded at: https://www.mdpi.com/article/10.3390/nu18132150/s1, Table S1: The 9 main compounds in Nagqu 4580.

## Abbreviations

The following abbreviations are used in this manuscript:

UC	Ulcerative colitis
AhR	Aryl hydrocarbon receptor
Nrf2	Nuclear factor erythroid 2-related factor 2
DSS	Dextran sulfate sodium
DAI	Disease activity index
MDA	Malondialdehyde
4-HNE	4-Hydroxynonenal
GSH	Glutathione
GSSG	Glutathione disulfide
GPX4	Glutathione peroxidase 4
IEC	Intestinal epithelial cell
IBD	Inflammatory bowel disease
5-HT	5-hydroxytryptamine
3-HA	3-hydroxyanthranilic acid
RTAs	Radical-trapping antioxidants
I3P	Indole-3-pyruvate
NCOA4	Nuclear receptor coactivator 4
FTH1	Ferritin heavy chain
PPP	Pentose phosphate pathway
GABA	Gamma-aminobutyric acid
LC-MS/MS	Lquid chromatography–tandem mass spectrometry
H&E	Hematoxylin and eosin
ZO-1	Zonula occludens-1
PCA	Principal component analysis
NAS	N-acetyltryptophan
IA	3-indoleacrylic acid
KYNA	Kynurenic acid
3-IS	3-indoxyl sulfate
5-HIAA	5-hydroxyindoleacetic acid
LEfSe	Linear discriminant analysis
