# Supplementary material for: Postbiotic Nagqu4580 Attenuates Ulcerative Colitis and Suppresses Ferroptosis in Association with the Microbiota-Tryptophan-AhR/Nrf2 Axis
Source: Nutrients. 2026 Jul 2;18(13):2150. doi: 10.3390/nu18132150 (PMC13363495; doi:10.3390/nu18132150)
Supplement: Supplementary file 1 [file nutrients-18-02150-s001.zip › nutrients-4337151-supplementary.pdf]

Table S1 The 9 main compounds in Nagqu 4580

No.	Compound Name	Rt（min）	Precursor m/z	Adduct Type	Formula
1	N-Acetyl-L-leucine	0.9099333	174.1125	$[M+H]^+$	C8H15NO3
2	N6-Methyl-2'-deoxyadenosine	0.92895	266.1234	$[M+H]^+$	C11H15N5O3
3	N-Acetylneuraminate	0.9883	292.1026	$[M+H]^+$	C11H19NO9
4	Isosakuranin	6.710633	449.1450	$[M+H]^+$	C22H24O10
5	Hesperidin	6.710633	611.1965	$[M+H]^+$	C28H34O15
6	Salvianolic acid B	6.796933	719.1603	[M+H]+	C36H30O16
7	Methoxy-myricetin-3-O-hexoside	7.164233	493.0988	$[M-H]^-$	C22H22O13
8	Phosphatidylinositol (16:1-18:2)	8.522817	831.5034	$[M-H]^-$	C43H77O13P
9	Stearamide	9.691033	284.2948	$[M+H]^+$	C18H37NO
